# Regulatory potential for concerted modulation of Nrf2- and Nfkb1-mediated gene expression in inflammation and carcinogenesis

**DOI:** 10.1038/sj.bjc.6604703

**Published:** 2008-12-02

**Authors:** S Nair, S T Doh, J Y Chan, A-N Kong, L Cai

**Affiliations:** 1 Department of Pharmaceutics, Rutgers, The State University of New Jersey, Piscataway, NJ 08854, USA; 2 Department of Biomedical Engineering, Rutgers, The State University of New Jersey, Piscataway, NJ 08854, USA; 3 Department of Pathology, University of California, Irvine, CA 92697, USA

**Keywords:** Nrf2, Nfkb1, inflammation, carcinogenesis

## Abstract

Many studies have implicated nuclear factor E2-related factor 2 (Nrf2) and nuclear factor-*κ*B1 (Nfkb1) in inflammation and cancer. However, the regulatory potential for crosstalk between these two important transcription factors in inflammation and carcinogenesis has not been explored. To delineate conserved transcription factor-binding site signatures, we performed bioinformatic analyses on the promoter regions of human and murine Nrf2 and Nfkb1. We performed multiple sequence alignment of Nrf2 and Nfkb1 genes in five mammalian species – human, chimpanzee, dog, mouse and rat – to explore conserved biological features. We constructed a canonical regulatory network for concerted modulation of Nrf2 and Nfkb1 involving several members of the mitogen-activated protein kinase (MAPK) family and present a putative model for concerted modulation of Nrf2 and Nfkb1 in inflammation/carcinogenesis. Our results reflect potential for putative crosstalk between Nrf2 and Nfkb1 modulated through the MAPK cascade that may influence inflammation-associated etiopathogenesis of cancer. Taken together, the elucidation of potential relationships between Nrf2 and Nfkb1 may help to better understand transcriptional regulation, as well as transcription factor networks, associated with the etiopathogenesis of inflammation and cancer.

The National Cancer Institute Inflammation and Cancer Think Tank in Cancer Biology (NCI, 2008) has recognised that epidemiological and clinical research corroborates an increased risk of certain cancers in the setting of chronic inflammation. Indeed, chronic inflammation, because of both infectious and non-infectious etiologies, has been associated with an increased risk of cancer development at a number of organ sites, with infectious agents estimated to be responsible for the development of 18% of all new cancer cases worldwide (Osburn *et al*, 2007). Infectious agents linked to cancer include hepatitis B virus and liver cancer, *Helicobacter pylori* and stomach cancer, and liver fluke infection and cholangiocarcinoma (Osburn *et al*, 2007). In addition, a number of inflammatory conditions without an infectious aetiology result in a significantly increased cancer risk as exemplified by chronic gastroesophageal reflux-induced oesophageal cancer, proliferative inflammatory atrophy-induced prostate cancer and chronic ulcerative colitis-associated colorectal cancer (Schottenfeld and Beebe-Dimmer, 2006; Osburn *et al*, 2007). Activation of inflammatory cells is accompanied by an increase in the release of reactive oxygen species (ROS) at the site of inflammation. Excess levels of ROS, due to chronic inflammation, may contribute to carcinogenesis by reacting with DNA to form oxidative DNA adducts possibly leading to mutagenesis and impaired regulation of cellular growth (Osburn *et al*, 2007). Many of the processes involved in inflammation (e.g., leukocyte migration, dilatation of local vasculature with increased permeability and blood flow and angiogenesis), when found in association with tumours, are more likely to contribute to tumour growth, progression and metastasis than to elicit an effective host antitumour response (NCI, 2008). Recently, it has been shown (Dougan and Dranoff, 2008) that signals downstream of the receptor for advanced glycation end products can fuel chronic inflammation, creating a microenvironment that is ideal for tumour formation in a mouse model of skin cancer.

Nuclear factor E2-related factor 2 (Nrf2 or Nfe2l2) is indispensable to cellular defense against many chemical insults of endogenous and exogenous origin, which play major roles in the etiopathogenesis of many cancers and inflammation-related diseases such as inflammatory bowel disease and Parkinson’s disease (Nair *et al*, 2007a). Under basal conditions, Nrf2 – a member of the Cap-N-Collar family of transcription factors – is sequestered in the cytoplasm by Keap1 resulting in enhanced proteasomal degradation of Nrf2. In conditions of oxidative stress, Nrf2 is released from Keap1 either by direct oxidative modification of Keap1 or after phosphorylation by redox-sensitive protein kinases, translocates to the nucleus and, in combination with other transcription factors, activates transcription of genes containing an antioxidant response element (ARE) in their promoter regions resulting in a cytoprotective adaptive response. This adaptive response is characterised by upregulation of a battery of antioxidative enzymes and decreased sensitivity to oxidative damage and cytotoxicity. These antioxidative enzymes have also been shown to attenuate inflammatory damage and neutralise ROS implicated in inflammatory signalling pathways. We have also reported (Khor *et al*, 2006) that Nrf2 could play an important role in protecting intestinal integrity, through the regulation of pro-inflammatory cytokines and induction of phase II detoxifying enzymes. Besides, we have demonstrated (Yuan *et al*, 2006) that mitogen-activated protein kinase (MAPK) pathways such as extracellular signal-regulated kinase (ERK) and c-jun N-terminal kinase (JNK) signalling pathways played important and positive roles in chemopreventive agent butylated hydroxyanisole-induced and Nrf2-dependent regulation of ARE-mediated gene expression, as well as the nuclear translocation of Nrf2 in HepG2 cells. We have observed earlier (Yu *et al*, 2000) that the activation of MAPK pathways induces ARE-mediated gene expression through a Nrf2-dependent mechanism. In addition, we have also shown (Shen *et al*, 2004) that different segments of the Nrf2 transactivation domain have different transactivation potentials and that different MAPKs have differential effects on Nrf2 transcriptional activity, with ERK and JNK pathways playing an unequivocal role in the positive regulation of Nrf2 transactivation domain activity (Shen *et al*, 2004; Nair *et al*, 2007a).

Importantly, recent mouse studies provide strong and direct genetic evidence that the classical IKK-*β* (inhibitor of nuclear factor-*κ*B (NF-*κ*B) kinase-*β*)-dependent NF-*κ*B activation pathway, which was proposed several years ago to be the molecular link between inflammation and carcinogenesis, is a crucial mediator of tumour promotion (Karin and Greten, 2005). Indeed, several pro-inflammatory cytokines and chemokines – such as tumour necrosis factor (TNF), IL-1, IL-6 and CXC-chemokine ligand 8 (CXCL8; also known as IL-8), all of which are encoded by the target genes of the IKK-*β*-dependent NF-*κ*B activation pathway – are associated with tumour development and progression in humans and mice. It has, thus, been hypothesised that activation of NF-*κ*B by the classical IKK-dependent pathway is a crucial mediator of inflammation-induced tumour growth and progression, as well as an important modulator of tumour surveillance and rejection (Karin and Greten, 2005). We have also demonstrated (Xu *et al*, 2005) that the suppression of NF-*κ*B and NF-*κ*B-regulated gene expression (VEGF, cyclin D1 and Bcl-XL) by chemopreventive isothiocyanates sulphoraphane and phenethyl isothiocyanate (PEITC) is mainly mediated through the inhibition of IKK phosphorylation, particularly IKK-*β*, and the inhibition of I*κ*B-*α* phosphorylation and degradation, as well as the decrease of nuclear translocation of p65 in human prostate cancer PC-3 cells. Recently, it has been observed (Murakami *et al*, 2007) that PEITC suppresses receptor activator of NF-*κ*B ligand-induced osteoclastogenesis by blocking the activation of ERK1/2 and p38 MAPK in RAW264.7 macrophages. Besides, HL60 cells treated with fisetin presented high expression of NF-*κ*B, activation of p38 MAPK and an increase of phosphoprotein levels (de Sousa *et al*, 2007).

Pro-inflammatory biomarkers such as IL-1*β*, IL-6, TNF-*α*, inducible nitric oxide synthase and cycloxygenase-2, which are all effector genes regulated by the NF-*κ*B pathway, have been noted (Li *et al*, 2008) to exhibit greater induction in Nrf2-deficient mice as compared with wild-type mice, indicating that ablation of Nrf2 seems to accelerate NF-*κ*B-mediated pro-inflammatory reactions. Besides, it has been shown recently (Liu *et al*, 2008) that NF-*κ*B competes with Nrf2 for binding to transcriptional coactivator CREB-binding protein and also promotes the recruitment of the corepressor histone deacetylase 3 to MafK leading to local histone hypoacetylation, thus, serving as a negative regulator of Nrf2–ARE signalling. Further, as constitutively active NF-*κ*B occurs in many inflammatory and tumour tissues (Liu *et al*, 2008), and as Nrf2 is implicated in the etiopathogenesis of many cancers and inflammation-associated conditions (Nair *et al*, 2007a), we elected to select these two important transcriptional regulators in this study to explore the potential for putative crosstalk between Nrf2 and NF-*κ*B signalling pathways in inflammation/injury and carcinogenesis. We performed *in silico* bioinformatic analyses to delineate conserved transcription factor-binding sites (TFBSs) or regulatory motifs in the promoter regions of human and murine Nrf2 and Nfkb1, as well as coregulated genes. We performed multiple sequence alignment of Nrf2 and Nfkb1 genes in five mammalian species and studied conserved biological features. We also looked at microarray data from public repositories such as Oncomine (Rhodes *et al*, 2005), Gene Expression Omnibus (GEO), Public Expression Profiling Resource (PEPR), as well as data sets from the Kong Laboratory, to dissect the role(s) of key regulatory genes in these selected inflammation/cancer signatures and constructed a regulatory network for concerted modulation of Nrf2 and Nfkb1 involving several members of the MAPK family. Our *in silico* analyses show that concerted modulation of Nrf2 and NF-*κ*B signalling pathways, and putative crosstalk involving multiple members of the MAPK family, may be potential molecular events governing inflammation and carcinogenesis.

## Materials and methods

### Identification of microarray data sets bearing inflammation/injury or cancer signatures

We perused several microarray data sets from public repositories such as Oncomine, GEO, PEPR, as well as data sets from the Kong Laboratory (Nair *et al*, 2006, 2007b, 2008). We selected 13 data sets that presented distinct signatures of inflammation or injury or carcinogenesis. Specifically, these studies reflected data on prostate cancer, spinal trauma, inflammatory response to injury and genes modulated by chemopreventive agents/toxicants in Nrf2-deficient animal models. These studies encompassed three mammalian species – human, mouse and rat – and exhibited modulation of both Nrf2 (Nfe2l2) and Nfkb1 genes as well as coregulated genes.

### Promoter analyses for transcription factor-binding sites

The promoter analyses were performed in the Cai Laboratory using Genomatix MatInspector (Quandt *et al*, 1995; Cartharius *et al*, 2005). Briefly, human promoter sequences of NFE2L2 and NFKB1, or corresponding murine promoter sequences, were retrieved from Gene2Promoter (Genomatix). Comparative promoter analyses were then performed by input of these sequences in FASTA format into MatInspector using optimised default matrix similarity thresholds. The similar and/or functionally related TFBSs were grouped into ‘matrix families,’ and graphical representations of common TFBS were generated. The ‘V$’ prefixes to the individual matrices are representative of the Vertebrate MatInspector matrix library. Similarly, we also elucidated common TFBS among the three topmost conserved human regulatory sequences after multiple alignment (as described below) of NRF2 and NFKB1 sequences.

### Multiple species alignment of Nrf2 (Nfe2l2) and Nfkb1 sequences

Non-coding sequences of Nfe2l2 and Nfkb1 genes in five mammalian species – human, chimpanzee, dog, mouse and rat – were retrieved using the Non-Coding Sequence Retrieval System (NCSRS) for comparative genomic analysis of gene regulatory elements that has been previously developed and published (Doh *et al*, 2007) by the Cai Laboratory, and is readily available at http://cell.rutgers.edu/ncsrs/. Multiple sequence alignment was performed by submitting the non-coding sequences to MLAGAN (Multi-LAGAN, Multi-Limited Area Global Alignment of Nucleotides) (Brudno *et al*, 2003), which is compatible with the VISTA visualisation tool. The MLAGAN alignments were, thus, visualised using VISTA by projecting them to pairwise alignments with respect to one reference sequence (human) as baseline. The common TFBSs between Nfe2l2 and Nfkb1 between the top biological features that were conserved in multiple species were then determined using Genomatix MatInspector as described earlier in Materials and Methods under Promoter Analyses for TFBS.

### Construction and validation of canonical first-generation regulatory network involving Nrf2 (Nfe2l2) and Nfkb1

A putative regulatory network for Nrf2 (Nfe2l2) and Nfkb1, representing 59 nodes and 253 potential interactions, was constructed using Cytoscape 2.5.2 software (Shannon *et al*, 2003). Further, we validated our network using PubGene (Jenssen *et al*, 2001), a literature network where connections are strong indicators of biological interaction. Additional validation was achieved by the generation of a biological network with these gene identifiers through the use of Ingenuity Pathways Analysis (Ingenuity Systems®; www.ingenuity.com). For this purpose, a data set containing gene identifiers was uploaded into the application. Each gene identifier was mapped to its corresponding gene object in the Ingenuity Pathways Knowledge Base. These genes, called focus genes, were overlaid onto a global molecular network developed from information contained in the Ingenuity Pathways Knowledge Base. Networks of these focus genes were then algorithmically generated based on their connectivity. In addition, we used dChip application (Li and Hung Wong, 2001; Li and Wong, 2001) to assess differential expression of MAPKs in cancer *vs* developmental or non-cancerous tissue/cell lines. Briefly, the CEL files created from each data set were first imported into dChip software for further data characterisation. A gene information file with current annotations and functional gene ontology was generated and the Affymetrix Chip Description File (CDF) was specified. The data were then normalised in dChip, and the expression value for each gene was determined by calculating the average of differences in intensity (perfect match intensity minus mismatch intensity) between its probe pairs. Finally, clustering and enrichment analysis was performed.

### A putative model for Nrf2–Nfkb1 interactions in inflammation and carcinogenesis

A pictorial model for Nrf2–Nfkb1 interactions was generated using Pathway Builder Tool 2.0 available from Protein Lounge, San Diego, CA, USA.

## Results

### Identification of microarray data sets bearing inflammation/injury or cancer signatures

To investigate distinct signatures of inflammation/injury or carcinogenesis, we perused several microarray data sets from public repositories such as Oncomine, GEO, PEPR, as well as microarray data sets from the Kong Laboratory. As summarised in Table 1, we selected 13 data sets that presented distinct signatures of inflammation/injury or carcinogenesis. Specifically, these studies reflected data on prostate cancer, spinal trauma, inflammatory response to injury and genes modulated by chemopreventive agents/toxicants in Nrf2-deficient animal models. These studies encompassed three mammalian species – human, mouse and rat – and exhibited modulation of both Nrf2 (or Nrf2-dependent) and Nfkb1 genes as well as coregulated genes. In other words, all these studies presented modulation of both Nrf2 (Nfe2l2) and Nfkb1 genes in concert, except for the studies with Nrf2-deficient animal models where Nrf2-dependent genes were elucidated. Interestingly, these data sets exhibited modulation of several key members of the MAPK family as well as cofactors of Nrf2 and Nfkb1. This literature pre-screen encouraged us to investigate further the regulatory potential for concerted modulation of Nrf2- and Nfkb1-mediated gene expression in inflammation and carcinogenesis using an *in silico* bioinformatic approach.

### Comparative promoter analyses of Nrf2 (Nfe2l2) and Nfkb1 for conserved transcription factor-binding sites

**Figure 1:**
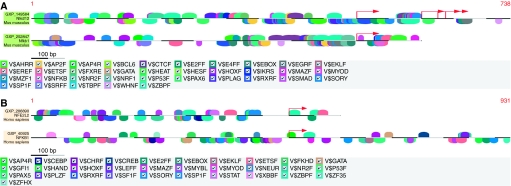
(**A**) A conserved TFBS between mouse Nfe2l2 and Nfkb1. Vertebrate (V$) matrix families conserved between murine Nfe2l2 and Nfkb1 promoter regions were identified using Genomatix MatInspector. (**B**) A conserved TFBS between human NFE2L2 and NFKB1. Vertebrate (V$) matrix families conserved between human NFE2L2 and NFKB1 promoter regions were identified using Genomatix MatInspector.

To identify conserved TFBS signatures, we performed comparative analyses of Nrf2 and Nfkb1 murine promoter sequences (Figure 1A) using Genomatix MatInspector (Quandt *et al*, 1995; Cartharius *et al*, 2005) as described in Materials and Methods. We also studied NRF2 and NFKB1 human promoter sequences (Figure 1B) similarly. Table 2 includes, as indicated, an alphabetical listing of the conserved vertebrate (V$) matrix families between these two transcription factors. The major human matrix families included activator protein 4 and related proteins, Ccaat/enhancer-binding protein, Camp-responsive element-binding proteins, E2F-myc activator/cell cycle regulator, E-box-binding factors, basic and erythroid krueppel-like factors, fork head domain factors, Myc-associated zinc fingers, nuclear receptor subfamily 2 factors, p53 tumour suppressor, RXR heterodimer-binding sites, SOX/SRY-sex/testis-determining and related HMG box factors, signal transducer and activator of transcription (STAT), X-box-binding factors, Zinc-binding protein factors and two-handed zinc finger homoeodomain transcription factors, among others. Some key conserved murine matrix families included AHR-arnt heterodimers and AHR-related factors, activator protein 2, activator protein 4 and related proteins, E2F-myc activator/cell cycle regulator, E-box-binding factors, basic and erythroid krueppel-like factors, farnesoid X-activated receptor response elements, heat-shock factors, Ikaros zinc finger family, Myc-associated zinc fingers, NF-*κ*B/c-rel, nuclear receptor subfamily 2 factors, nuclear respiratory factor 1, p53 tumour suppressor, pleomorphic adenoma gene, RXR heterodimer-binding sites, SOX/SRY-sex/testis-determining and related HMG box factors, serum response element-binding factor, Tata-binding protein factor, zinc-binding protein factors, among others. Indeed, as evident from Table 2, several matrix families were conserved between Nrf2 and Nfkb1 in both human and murine promoters.

### Multiple species alignment of Nrf2 (Nfe2l2) and Nfkb1 sequences

**Figure 2:**
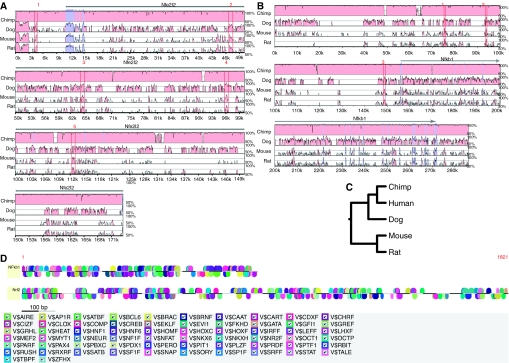
Multiple species alignment. Non-coding sequences of Nfe2l2 and Nfkb1 genes in five mammalian species – human, chimpanzee, dog, mouse and rat – were retrieved using the Non-Coding Sequence Retrieval System (NCSRS) for comparative genomic analysis of gene regulatory elements. Multiple sequence alignment was performed by submitting the non-coding sequences to MLAGAN and visualised by projecting them to pairwise alignments with respect to one reference sequence (human) as baseline. Pink regions, conserved non-coding sequences (CNS); dark blue regions, exons. The numbers indicate CNS that were identified across species. (**A)** Multiple species alignment for Nfe2l2; (**B**) multiple species alignment for Nfkb1; (**C**) phylogenetic tree for Nfe2l2 and Nfkb1; (**D**) a conserved TFBS between NFE2L2 and NFKB1 among top matching human sequences.

With the objective of investigating conserved biological features across different mammalian species, we performed multiple species alignment of Nrf2 (Nfe2l2) and Nfkb1 sequences as described in Materials and Methods. We used NCSRS for comparative genomic analysis of gene regulatory elements that has been previously developed and published (Doh *et al*, 2007), and is readily available at http://cell.rutgers.edu/ncsrs/ and retrieved non-coding sequences of Nfe2l2 and Nfkb1 genes in five mammalian species – human, chimpanzee, dog, mouse and rat. As shown in Figures 2A and B, we performed multiple sequence alignment using MLAGAN (Brudno *et al*, 2003) for Nfe2l2 and Nfkb1 genes, respectively, with respect to one reference sequence (human) as baseline. The phylogenetic tree for Nfe2l2 and Nfkb1 in the five species under consideration was constructed (Figure 2C). The conserved biological features across species for each of Nfe2l2 and Nfkb1 genes were perused, and the top five features for Nfe2l2 and the top three features for Nfkb1, as numbered in Figures 2A and B, are listed in Tables 3A and B, respectively. Sequence 4 for Nfe2l2 and sequence 1 for Nfkb1 exhibited the highest degree of conservation across species at 98.86 and 86.58%, respectively. In addition, the top three conserved sequences in the human sequences of both these genes (sequences 4, 3 and 2 for Nfe2l2 in that order and sequences 1, 2 and 3 for Nfkb1 in that order) as evident from Tables 3A and B were submitted to Genomatix MatInspector. The common TFBSs between Nfe2l2 and Nfkb1 between these biological features that were conserved in multiple species were then determined (Figure 2D) and tabulated along with the other TFBS results for comparative promoter analyses in Table 2 discussed earlier.

### Construction and validation of a canonical first-generation regulatory network involving Nrf2 (Nfe2l2) and Nfkb1

**Figure 3:**
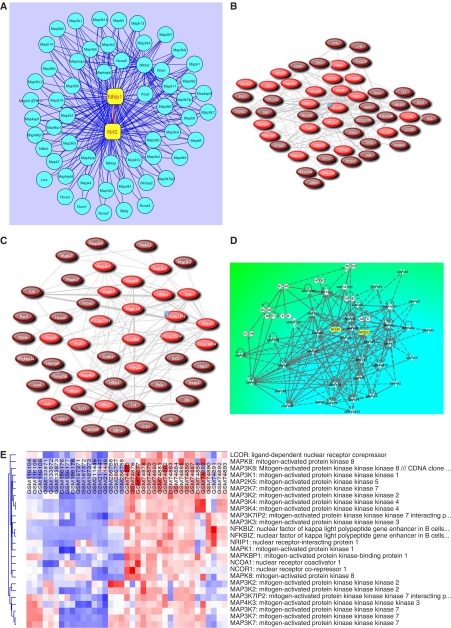
A canonical regulatory network for Nrf2–Nfkb1 interactions in inflammation-associated carcinogenesis. (**A**) A putative regulatory network for Nrf2 (Nfe2l2) and Nfkb1 representing 59 nodes and 253 potential interactions implicating several members of the MAPK family; (**B**) literature network in humans; (**C**) literature network in mice; (**D**) functional crosstalk in biological network of Nfe2l2, Nfkb1 and various members of the MAPK cascade; (**E**) differential expression of MAPKs in cancer *vs* developmental or non-cancerous tissue/cell lines (GSM116104–116106: bronchial smooth muscle cells; GSM133871–133873: retinal pigment epithelial cell line; GSM156176–156178: skeletal muscle; GSM187371–187373: untreated LNCaP cells; GSM211446–211448: normal adrenal gland; GSM286756–286758: untreated MCF7 cells; GSM74875–74880: benign prostate tissue; GSM74881–74887: clinically localised primary prostate cancer; GSM74888–74893: metastatic prostate cancer).

To construct a canonical first-generation biological network for Nrf2–Nfkb1 interactions, we streamlined our study to five data sets summarised in Table 4, which were representative of the most distinct inflammation/injury and cancer signatures from the 13 data sets perused earlier. We obtained gene expression values for 59 genes, as shown in Table 4, including Nrf2 (Nfe2l2), Nfkb1, several cofactors and many members of the MAPK family from these data sets. As shown in Figure 3A, we constructed a putative first-generation regulatory network for Nrf2 (Nfe2l2) and Nfkb1, representing 59 nodes and 253 potential interactions using Cytoscape 2.5.2 software (Shannon *et al*, 2003). Further, we validated our network by submitting 20 representative genes from Table 4 to PubGene (Jenssen *et al*, 2001), a literature network where connections are strong indicators of biological interaction, and retrieved biological networks in human (Figure 3B) and mouse (Figure 3C), thus delineating gene signatures that validated the putative biological role(s) of the genes elucidated in this *in silico* study that served as the source and target nodes in our regulatory network. We further validated our network by querying Ingenuity Pathways Analysis (Ingenuity Systems; www.ingenuity.com) application with the 59 gene identifiers forming the basis of our network and obtained a biological network (Figure 3D) with a high degree of functional crosstalk based on the Ingenuity Knowledge Base reiterating the potential for crosstalk between multiple members of the MAPK family that might modulate the Nrf2–Nfkb1 interactions as indicated in our canonical network (Figure 3A). Furthermore, we used dChip application (Li and Hung Wong, 2001; Li and Wong, 2001) to assess the differential expression of MAPKs in cancerous *vs* developmental or non-cancerous tissue/cell lines by using several unrelated microarray data sets from the GEO resource at the NCBI including GSM116104–116106: bronchial smooth muscle cells; GSM133871-133873: retinal pigment epithelial cell line; GSM156176–156178: skeletal muscle; GSM187371–187373: untreated LNCaP cells; GSM211446–211448: normal adrenal gland; GSM286756–286758: untreated MCF7 cells; GSM74875–74880: benign prostate tissue; GSM74881–74887: clinically localised primary prostate cancer; and GSM74888–74893: metastatic prostate cancer. Results from this analysis (Figure 3E) show the differential expression of several MAPKs in cancer *vs* non-cancerous tissue.

### A putative model for Nrf2–Nfkb1 interactions in inflammation and carcinogenesis

**Figure 4:**
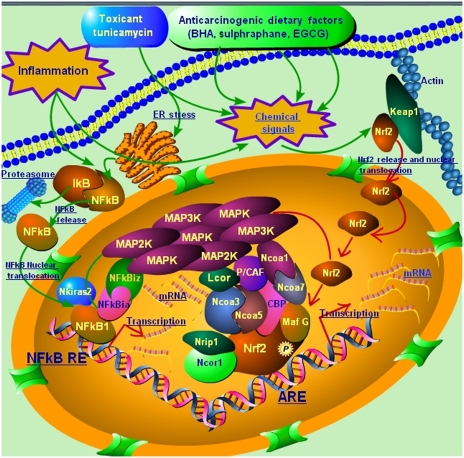
A putative model for Nrf2-–Nfkb1 interactions in inflammation and carcinogenesis. Chemical signals generated by dietary chemopreventive agents or toxicants, or inflammatory signals, may cause Nrf2 nuclear translocation that sets in motion a dynamic machinery of coactivators and corepressors that may form a multimolecular complex with Nrf2 for modulating transcriptional response through the antioxidant response element, ARE. Inflammation may also cause release of NF-*κ*B from I*κ*B and stimulate NF-*κ*B nuclear translocation to modulate transcriptional response through the NF-*κ*B response element, NF-*κ*B-RE, along with the cofactors of NF-*κ*B. Several members of the MAPK family may act in concert with Nrf2 and Nfkb1 with multiple interactions between the members of the putative complex to elicit the chemopreventive and pharmacotoxicological events in inflammation and carcinogenesis.

On the basis of the extensive experience (Yu *et al*, 2000; Li *et al*, 2005, 2006; Yuan *et al*, 2006; Nair *et al*, 2007a; Prawan *et al*, 2008) of the Kong Laboratory with Nrf2–Keap1 pathway and role(s) of MAPK/dietary chemopreventives/toxicants, our many microarray studies (Shen *et al*, 2005, 2006; Keum *et al*, 2006; Nair *et al*, 2006, 2007b, 2008; Hu *et al*, 2006a, 2006b; Barve *et al*, 2008) in Nrf2-deficient mice, our studies (Jeong *et al*, 2004; Xu *et al*, 2005) on NF-*κ*B pathway and chemopreventive agents, and the gene signatures elicited in inflammation and carcinogenesis in this *in silico* study using our data as well as publicly available data from other research groups as indicated earlier, we generated a pictorial model (Figure 4) for Nrf2–Nfkb1 interactions using Pathway Builder Tool 2.0. available from Protein Lounge, San Diego, CA. In essence, chemical signals generated by dietary chemopreventive agents or toxicants, or inflammatory signals, may cause Nrf2 nuclear translocation that sets in motion a dynamic machinery of coactivators and corepressors that may form a multi-molecular complex with Nrf2 to modulate transcriptional response through the ARE. Inflammation may also cause release of NF-*κ*b1 from I*κ*B and stimulate NF-*κ*b1 nuclear translocation to modulate transcriptional response through the NF-*κ*b1 response element, NF-*κ*b-RE, along with cofactors of NF-*κ*b1. Several members of the MAPK family may act in concert with Nrf2 and Nfkb1 with multiple interactions between the members of the putative complex to elicit the chemopreventive and pharmacotoxicological events in inflammation and carcinogenesis.

## Discussion

Karin and Greten (2005) succinctly noted that carcinogenesis may be divided into three mechanistic phases: initiation (which involves stable genomic alterations), promotion (which involves the proliferation of genetically altered cells) and progression (which involves an increase in the size of the tumour, the spreading of the tumour and the acquisition of additional genetic changes). In 1863, Rudolf Virchow observed leukocytes in neoplastic tissues and suggested (Balkwill and Mantovani, 2001) that the ‘lymphoreticular infiltrate’ reflected the origin of cancer at sites of chronic inflammation. Besides, persistent and recurrent episodes of inflammation (McCulloch *et al*, 2006), mediated by aberrant activation of innate and acquired immunity, characterise a wide spectrum of idiopathic and infectious chronic inflammatory disorders. In response to tissue injury (Coussens and Werb, 2002), a multifactorial network of chemical signals initiate and maintain a host response designed to ‘heal’ the afflicted tissue involving activation and directed migration of leukocytes (neutrophils, monocytes and eosinophils) from the venous system to sites of damage, and tissue mast cells also play a significant role. Interestingly, inflammation and innate immunity most commonly exert pro-tumorigenic effects (Karin and Greten, 2005) mediated through different types of leukocytes, including normal tissue macrophages, tumour-associated macrophages, dendritic cells, neutrophils, mast cells and T cells, which are recruited to the tumour microenvironment through interactions with local stromal cells and malignant cells. These leukocytes produce cytokines, and growth and angiogenic factors, as well as matrix-degrading proteases (such as the matrix metalloproteinases MMP1, MMP3 and MMP9) and their inhibitors, which allow tumour cells to proliferate, invade and metastasise (Karin and Greten, 2005). Although the causal relationship between inflammation, innate immunity and cancer is more widely accepted, many of the molecular and cellular mechanisms mediating this relationship remain unresolved (Coussens and Werb, 2002). Many studies have implicated Nrf2 (Nfe2l2) in cancer (Ramos-Gomez *et al*, 2001; Katsuoka *et al*, 2005; Sporn and Liby, 2005; Nair *et al*, 2007a; Pearson *et al*, 2008) or inflammation-associated diseases such as colitis (Khor *et al*, 2006; Osburn *et al*, 2007), and Parkinson's disease (Clements *et al*, 2006), and NF-*κ*B in inflammation (Muller-Ladner *et al*, 2002; Profita *et al*, 2008; Puthia *et al*, 2008) and cancer (Xu *et al*, 2005; Cilloni *et al*, 2006; Sun and Zhang, 2007; McDonnell *et al*, 2008). Indeed, the identification of combinatorial, or synergistic, transcription factors and the elucidation of relationships among them are of great importance for understanding transcriptional regulation as well as transcription factor networks (Hu *et al*, 2007). However, despite a growing recognition of the important role(s) played by these two pivotal transcription factors, the regulatory potential for crosstalk between these two important transcription factors in inflammation and carcinogenesis has not been explored.

The perusal of several microarray data sets from public repositories such as Oncomine, GEO, PEPR, as well as data sets from the Kong Laboratory, facilitated the identification of 13 data sets (Table 1) presenting distinct signatures of inflammation/injury or carcinogenesis that served as our literature pre-screen for concerted modulation of Nrf2 and Nfkb1 genes. The comparative analyses of TFBS in these two gene promoters revealed that many matrix families were conserved between human NRF2 and NFKB1 promoters, and between murine Nrf2 and Nfkb1 promoter regions (Figure 1 and Table 2). Furthermore, as elucidated in Table 2, several functionally important matrix families were also found to be common across human and murine species, including activator protein 4, E2F-myc activator/cell cycle regulators (V$E2FF), E-box-binding factors, basic and erythroid krueppel-like factors, p53 tumour suppressor and RXR heterodimer-binding sites (V$RXRF), among others. The identification of V$E2FF is significant because disruption of retinoblastoma protein, a key controller of E2F activity and G1/S transition in the cell cycle, can alter the growth-inhibitory potential of TGF-*β* in the inflammatory milieu of chronic liver disease and contribute to cancer development (Sheahan *et al*, 2007). Inflammatory conditions can enhance the genotoxic effects of carcinogenic polycyclic aromatic hydrocarbons such as benzo[a]pyrene (BaP) through the upregulation of CYP1B1 expression associated with increased phosphorylation of p53 tumour suppressor at Ser-15 residue, enhanced accumulation of cells in the S-phase of the cell cycle and potentiation of BaP-induced apoptosis (Umannova *et al*, 2008). Thus, the presence of conserved p53 TFBS in Nrf2/Nfkb1 promoters may point to a critical role for inflammation in the etiopathogenesis of cancer and underscore the relevance of crosstalk between these two transcription factors. The identification of V$RXRF is important as RXR physically interacts with peroxisome proliferator-activated receptor (PPAR-*α*), a major player in lipid metabolism and inflammation, and PPAR-*α* agonists such as fenofibrate inhibit NF-*κ*B DNA-binding activity (Xu *et al*, 2006). In addition, a conserved TFBS for NF-*κ*B itself (Table 2) was found to be present in murine promoter regions of Nrf2 and Nfkb1, strengthening the potential for crosstalk between these two transcription factors. Our multiple sequence alignments (Figures 2A and B and Tables 3A and B) enabled the study of conserved biological features for each gene across five mammalian species and the construction of a phylogenetic tree (Figure 2C). Interestingly, several key biological features were elicited on subjecting the top three conserved human sequences of each gene to comparative promoter analyses (Figure 2D and Table 2). Notably, autoimmune regulatory element-binding factors (V$AIRE) and PPAR (or V$PERO) were conserved in these promoters. Recently, a cyclopentenonic prostaglandin 15-deoxy-delta(12,14)-prostaglandin J(2) has been shown to inhibit TNF-related apoptosis-inducing ligand mRNA expression by downregulating the activity of its promoter in T lymphocytes, with NF-*κ*B being identified as a direct target of this prostanoid that is also regulated by the activation of PPAR-*γ* (Fionda *et al*, 2007). The identification of PPAR in the promoter regions of Nrf2 and Nfkb1 in this study, thus, reinforces the significance of these transcription factors and provides a possible mechanistic pathway for crosstalk in inflammation and cancer. Interestingly, a recent study (Kim *et al*, 2008) has reported that treatment of human brain astrocytes with double-stranded RNA induced interferon regulatory factor 3 (IRF3) phosphorylation and nuclear translocation followed by activation of STAT1 along with a concomitant activation of NF-*κ*B and MAPK cascade members (p38, JNK and ERK). In this study, we identified interferon regulatory factors (IRFF) and STAT as being conserved in Nrf2 and Nfkb1 promoters, as well as MAPK members in our regulatory network (Figure 3A), which agrees with the mechanistic evidence from the brain astrocyte study. It has been observed (Tliba *et al*, 2006) that stimulation with pro-inflammatory cytokines of CD38, known to be responsible for lung airway inflammation, rendered it insensitive to treatment with glucocorticoids such as fluticasone, dexamethasone or budesonide, by inhibiting steroid-induced glucocorticoid-responsive element (GRE)-dependent gene transcription. We also identified conserved GRE in the Nrf2/Nfkb1 promoters that further validated our results as biologically relevant. In addition, cell cycle regulators, heat-shock factors and several other matrices were found to be conserved between the two genes, thus, underscoring the biological relevance, and the intrinsic complexity, of Nrf2/Nfkb1 crosstalk from a functional standpoint.

Further, we streamlined our study to five data sets (Table 4), which were representative of the most distinct inflammation/injury and cancer signatures of interest and constructed a canonical first-generation regulatory network (Figure 3A) for Nrf2 (Nfe2l2) and Nfkb1, representing 59 nodes and 253 potential interactions. We generated functionally relevant PubGene literature networks in human (Figure 3B) and mouse (Figure 3C), using the Ingenuity Knowledge Base (Figure 3D), thus delineating gene signatures that validated the biological role(s), and potential for crosstalk, of the genes elucidated in this *in silico* study. We also assessed the expression of MAPKs in several randomly picked, unrelated microarray data sets of cancerous and non-cancerous origin and showed that various MAPKs are differentially expressed in cancer *vs* developmental or non-cancerous tissue/cell lines (Figure 3E). Our future study includes the expansion of our study objectives to generate more detailed second-generation or third-generation regulatory networks for Nrf2 and Nfkb1 as more functional data emerge on these gene targets of interest and their interactions with coactivator/corepressor modules that associate with them. Interestingly, as shown in our current first-generation network (Figure 3A), several MAPKs play a central role in mediating the transcriptional effects of Nrf2 and Nfkb1. This is, indeed, in consonance with the known role of MAPKs in potentiating Nrf2-mediated ARE activation (Yu *et al*, 2000; Shen *et al*, 2004; Yuan *et al*, 2006) and their role in modulating NF-*κ*B (Murakami *et al*, 2007; de Sousa *et al*, 2007), thus, further underscoring the biological applicability of our results. Finally, we present a gestalt pictorial overview (Figure 4) of our current knowledge of concerted modulation of Nrf2 and Nfkb1 based on the data from this study and our extensive experience in cancer chemoprevention.

The results from our current *in silico* study may strengthen the possibility that scientists could, in the future, consider pursuing Nrf2, Nfkb1 and MAPKs as potential targets in early drug discovery screens for the management of inflammation and cancer. In contemporary times, systems biology has interfaced with the drug discovery process to enable high-throughput screening of multiple drug targets and target-based leads. Indeed, a combination of high-throughput screening, kinase-specific libraries and structure-based drug design has facilitated the discovery of selective kinase inhibitors (Manning and Davis, 2003). Needless to add, the benefits of applying molecular profiling to drug discovery and development include much lower failure rates at all stages of the drug development pipeline, faster progression from discovery through to clinical trials and more successful therapies for patient subgroups (Stoughton and Friend, 2005). Thus, the development of specific inhibitors that might regulate the specific crosstalk between the two central pleiotropic transcription factors Nrf2 and Nfkb1, and with the associated family of kinases, may be one of many strategies that might aid in the drug discovery process. Taken together, our study provides a canonical framework to understand the regulatory potential for concerted modulation of Nrf2 and Nfkb1 in inflammation and cancer. Further studies addressing this question with specific emphasis on cofactor modules binding to these transcription factors and coregulation with upstream signalling molecules in the MAPK cascade will enable a better appreciation of the emerging key role(s) of, and the crosstalk between, these two transcription factors in inflammation and carcinogenesis.

## Figures and Tables

**Table 1:** Microarray data sets bearing inflammation/injury or cancer signatures

**Species**	**Tissue**	**Study**	**Data source**	**Descriptor**	**Affymetrix platform**
Human	Prostate	Lapointe	Oncomine	Prostate cancer	Non-Affymetrix
Human	Prostate	Luo	Oncomine	Prostate cancer and benign prostatic hyperplasia	Non-Affymetrix
Mouse	Lung	Kleeberger	GEO	Hyperoxic lung injury	MG U74Av2
Mouse	Lung	Papaiahgari	GEO	Lung injury and inflammatory response	MG 430A 2.0
Mouse	Spleen and liver	Li	GEO	Autoimmune disease and Nrf2	MG U74Av2
Mouse	Type II cells	Machireddy	GEO	Nrf2 wild-type and knockout cells	MG 430 2.0
Mouse	Prostate	Nair 1	Kong Laboratory	EGCG+SFN combination treatment	MG 430 2.0
Mouse	Small intestine and liver	Nair 2	Kong Laboratory	BHA treatment	MG 430 2.0
Mouse	Small intestine and liver	Nair 3	Kong Laboratory	Induction of ER stress with TM	MG 430 2.0
Rat	Spinal cord	Faden 1	PEPR	Supraspinal tracts	RG_U34A
Rat	Spinal cord	Faden 2	PEPR	Trauma above T9	RG_U34A
Rat	Spinal cord	Faden 3	PEPR	Trauma below T9	RG_U34A
Rat	Spinal cord	Faden 4	PEPR	Trauma T9	RG_U34A

BHA=butylated hydroxyanisole; EGCG=epigallocatechin-3-gallate; ER=endoplasmic reticulum; SFN=sulphoraphane; TM=tunicamycin.

All Nair data sets are from the Kong Laboratory as discussed in Materials and Methods; all Faden data sets are as defined by descriptors detailed above at the PEPR resource; all other data sets are as defined by descriptors detailed above at the Oncomine or GEO resources.

**Table 2:** Human and murine matrix families conserved between Nrf2 and Nfkb1

**Matrix family (Human NRF2 *vs* NFKB1)**	**Matrix family (Murine Nrf2 *vs* Nfkb1)**	**Matrix family conserved after multiple species alignment (NRF2 *vs* NFKB1)**	**Family information**
–	V$AHRR	–	AHR-arnt heterodimers and AHR-related factors
–	–	V$AIRE	Autoimmune regulatory element-binding factor
–	–	V$AP1R	MAF- and AP1-related factors
–	V$AP2F	–	Activator protein 2
V$AP4R	V$AP4R	–	Activator protein 4 and related proteins
–	–	V$ATBF	AT-binding transcription factor
–	V$BCL6	V$BCL6	POZ domain zinc finger expressed in B-cells
–	–	V$BRAC	Brachyury gene, mesoderm developmental factor
–	–	V$BRNF	Brn POU domain factors
–	–	V$CAAT	CCAAT-binding factors
–	–	V$CART	Cart-1 (cartilage homoeoprotein 1)
–	–	V$CDXF	Vertebrate caudal related homoeodomain protein
V$CEBP	–	–	Ccaat/enhancer-binding protein
V$CHRF	–	V$CHRF	Cell cycle regulators: cell cycle homology element
–	–	V$CIZF	CAS-interating zinc finger protein
–	–	V$CLOX	CLOX and CLOX homology (CDP) factors
–	–	V$COMP	Factors that cooperate with myogenic proteins
V$CREB	–	V$CREB	Camp-responsive element-binding proteins
–	V$CTCF	–	CTCF and BORIS gene family, transcriptional regulators with 11 highly conserved zinc finger domains
V$E2FF	V$E2FF	–	E2F-myc activator/cell cycle regulator
–	V$E4FF	–	Ubiquitous GLI-krueppel-like zinc finger involved in cell cycle regulation
V$EBOX	V$EBOX	–	E-box-binding factors
–	V$EGRF	–	EGR/nerve growth factor-induced protein C and related factors
V$EKLF	V$EKLF	V$EKLF	Basic and erythroid krueppel-like factors
–	V$EREF	–	Estrogen response elements
V$ETSF	V$ETSF	–	Human and murine ETS1 factors
–	–	V$EVI1	EVI1 myeloid-transforming protein
V$FKHD	–	V$FKHD	Fork head domain factors
–	V$FXRE	–	Farnesoid X-activated receptor response elements
V$GATA	V$GATA	V$GATA	GATA-binding factors
V$GFI1	–	V$GFI1	Growth factor independence transcriptional repressor
–	–	V$GREF	Glucocorticoid responsive and related elements
–	–	V$GRHL	Grainyhead-like transcription factors
V$HAND	–	–	bHLH transcription factor dimer of HAND2 and E12
–	V$HEAT	V$HEAT	Heat-shock factors
–	V$HESF	–	Vertebrate homologues of enhancer of split complex
–	–	V$HNF1	Hepatic nuclear factor 1
–	–	V$HNF6	Onecut homoeodomain factor HNF6
–	–	V$HOMF	Homoeodomain transcription factors
–	–	V$HOXC	HOX–PBX complexes
V$HOXF	V$HOXF	V$HOXF	Factors with moderate activity to homoeodomain consensus sequence
–	V$IKRS	–	Ikaros zinc finger family
–	–	V$IRFF	Interferon regulatory factors
V$LEFF	–	V$LEFF	LEF1/TCF, involved in the Wnt signal transduction pathway
–	–	V$LHXF	Lim homoeodomain factors
V$MAZF	V$MAZF	–	Myc-associated zinc fingers
–	–	V$MEF2	MEF2, myocyte-specific enhancer-binding factor
V$MYBL	–	–	Cellular and viral myb-like transcriptional regulators
V$MYOD	V$MYOD	–	Myoblast-determining factors
–	–	V$MYT1	MYT1 C2HC zinc finger protein
–	V$MZF1	–	Myeloid zinc finger 1 factors
V$NEUR	–	V$NEUR	NeuroD, *β*2, HLH domain
–	–	V$NF1F	Nuclear factor 1
–	–	V$NFAT	Nuclear factor of activated T cells
–	V$NFKB	–	Nuclear factor *κ* B/c-rel
–	–	V$NKX6	NK6 homoeobox transcription factors
–	–	V$NKXH	NKX homoeodomain factors
V$NR2F	V$NR2F	V$NR2F	Nuclear receptor subfamily 2 factors
–	V$NRF1	–	Nuclear respiratory factor 1
–	–	V$OCT1	Octamer-binding protein
–	–	V$OCTP	OCT1-binding factor (POU-specific domain)
V$P53F	V$P53F	–	p53 tumour suppressor
–	–	V$PARF	PAR/bZIP family
V$PAX5	–	–	PAX-5 B cell-specific activator protein
–	V$PAX6	V$PAX6	PAX-4/PAX-6 paired domain-binding sites
–	–	V$PBXC	PBX1–MEIS1 complexes
–	–	V$PDX1	Pancreatic and intestinal homoeodomain transcription factor
–	–	V$PERO	Peroxisome proliferator-activated receptor
–	–	V$PIT1	GHF-1 pituitary-specific pou domain transcription factor
–	V$PLAG	–	Pleomorphic adenoma gene
V$PLZF	–	V$PLZF	C2H2 zinc finger protein PLZF
–	–	V$PRDF	Positive regulatory domain I-binding factor
–	–	V$PTF1	Pancreas transcription factor 1, heterotrimeric transcription factor
–	–	V$RBIT	Regulator of B-cell IgH transcription
–	–	V$RUSH	SWI/SNF-related nucleophosphoproteins with a RING finger DNA-binding motif
V$RXRF	V$RXRF	V$RXRF	RXR heterodimer-binding sites
–	–	V$SATB	Special AT-rich sequence-binding protein
V$SF1F	–	V$SF1F	Vertebrate steroidogenic factor
–	V$SMAD	–	Vertebrate SMAD family of transcription factors
–	–	V$SNAP	snRNA-activating protein complex
V$SORY	V$SORY	V$SORY	SOX/SRY-sex/testis-determining and related HMG box factors
V$SP1F	V$SP1F	V$SP1F	GC-Box factors SP1/GC
–	V$SRFF	V$SRFF	Serum response element-binding factor
V$STAT	–	V$STAT	Signal transducer and activator of transcription
–	–	V$TALE	TALE homoeodomain class-recognising TG motifs
–	V$TBPF	V$TBPF	Tata-binding protein factor
–	V$WHNF	–	Winged helix-binding sites
V$XBBF	–	–	X-box-binding factors
V$ZBPF	V$ZBPF	–	Zinc-binding protein factors
V$ZF35	–	–	Zinc finger protein ZNF35
V$ZFHX	–	V$ZFHX	Two-handed zinc finger homoeodomain transcription factors

**Table 3:** (A) Multiple species alignment for Nfe2l2. (B) Multiple species alignment for Nfkb1

**Nfe2l2**		**CNS start**	**CNS end**	**% id**	**Location**	**Length**	**Score**	**Chr**	**Strand**	**Start**	**End**
**(A)**											
	1			84.2		200.50	87.57				
Human		4424	4568	–	Intergenic	144		2	−	177796698	177796842
Chimpanzee		1559	12467	98.4	Intergenic			2b	−	182248910	182259818
Dog		4037	4426	78.9	Intergenic	389		36	−	24010936	24011325
Mouse		3993	4131	79.6	Intergenic	138		2	−	75470119	75470257
Rat		4002	4133	80.0	Intergenic	131		3	−	58360804	58360935
	2			79.9		801.25	93.25				
Human		46618	47576	–	Intronic	958		2	−	177838892	177839850
Chimpanzee		16433	72741	98.0	Intronic			2b	−	182263784	182320092
Dog		44082	45048	76.3	Intronic	966		36	−	24050981	24051947
Mouse		49275	49911	73.0	Intronic	636		2	−	75515401	75516037
Rat		48247	48892	72.3	Intronic	645		3	−	58405049	58405694
	3			81.6		746.50	94.04				
Human		63951	64978	–	Intronic	1027		2	−	177856225	177857252
Chimpanzee		16433	72741	98.0	Intronic			2b	−	182263784	182320092
Dog		63914	64953	78.0	Intronic	1039		36	−	24070813	24071852
Mouse		69040	69553	74.9	Intronic	513		2	−	75535166	75535679
Rat		68447	68854	75.5	Intronic	407		3	−	58425249	58425656
	4			82.8		962.25	98.86				
Human		95699	97201	–	Intronic	1502		2	−	177887973	177889475
Chimpanzee		93603	105622	98.3	Intronic			2b	−	182340954	182352973
Dog		94052	95563	80.9	Intronic	1511		36	−	24100951	24102462
Mouse		99967	100384	76.4	Intronic	417		2	−	75566093	75566510
Rat		103210	103629	75.7	Intronic	419		3	−	58460012	58460431
	5			82.8		418.75	89.74				
Human		112361	112533	–	Intronic	172		2	−	177904635	177904807
Chimpanzee		113121	117879	98.1	Intronic			2b	−	182360472	182365230
Dog		105905	106887	82.1	Intronic	517		36	−	24112804	24113786
Mouse		115077	115617	74.8	Intronic	540		2	−	75581203	75581743
Rat		119448	119894	76.1	Intronic	446		3	−	58476250	58476696
											
**(B)**											
	1			78		491	86.58				
Human		75272	75516	–	Intergenic	244		4	+	103675774	103675545
Rat		190119	189890	77	Intergenic	229		2	−	233663290	233663525
Mouse		585736	585501	80	Intergenic	235		3	−	135287512	135286504
Dog		52140	53148	78	Intergenic	1008		32	+	26791395	26841008
Chimpanzee		67709	117322	98	Intergenic	49613		4	+	105654198	105654198
	2			80		319	84.82				
Human		93740	94016	–	Intergenic	276		4	+	103659473	103659195
Rat		173818	173540	79	Intergenic	278		2	−	233651850	233652129
Mouse		574340	574061	78	Intergenic	279		3	−	135307030	135306630
Dog		72266	72666	81	Intergenic	400		32	+	26791395	26841008
Chimpanzee		67709	117322	98	Intergenic	49613		4	+	105654198	105654198
	3			74		469	81.92				
Human		148427	148852	–	Intergenic	425		4	+	103626900	103626486
Rat		141245	140831	73	Intergenic	414		2	−	233620618	233620924
Mouse		543135	542829	73	Intergenic	306		3	−	135342413	135341726
Dog		107362	108049	76	Intergenic	687		32	+	26841253	26877988
Chimpanzee		117567	154302	99	Intergenic	36735		4	+	105654198	105654198

Chr=chromosome; CNS=conserved non-coding sequences.

**Table 4:** Canonical first-generation regulatory network members representing putative crosstalk between Nrf2 (Nfe2l2) and Nfkb1 in inflammation-associated carcinogenesis^a^

**Sr. no.**	**GenBank accession no.**	**Gene name**	**Gene symbol**	**Papaiahgari lung injury and inflammation**	**SFN+EGCG Nrf2-dependent genes**	**BHA Nrf2- dependent genes**	**Tunicamycin Nrf2-dependent genes**	**Faden supraspinal tracts**
1	NM_172154	Ligand-dependent nuclear receptor corepressor	Lcor	–	−3.25	–	–	–
2	NM_010756	v-maf musculoaponeurotic fibrosarcoma oncogene family, protein G (avian)	Mafg	–	–	2.45	–	–
3	NM_008927	Mitogen-activated protein kinase kinase 1	Map2k1	−2.7	−4.12	2.28	–	1.59
4	NM_023138	Mitogen-activated protein kinase kinase 2	Map2k2	–	–	2.75	1.15	1.1
5	NM_009157	Mitogen-activated protein kinase kinase 4	Map2k4	−1.55	−3.31	–	−1.68	–
6	NM_011840	Mitogen-activated protein kinase kinase 5	Map2k5	−1.56	–	−1.45	–	1.59
7	NM_011943	Mitogen-activated protein kinase kinase 6	Map2k6	–	−3.98	–	–	1.19
8	NM_011944	Mitogen-activated protein kinase kinase 7	Map2k7	−1.32	–	−0.37	−0.16	–
9	NM_011945	Mitogen-activated protein kinase kinase kinase 1	Map3k1	–	–	−0.28	−0.18	1.11
10	NM_011946	Mitogen-activated protein kinase kinase kinase 2	Map3k2	–	−5.02	–	2.25	–
11	NM_011947	Mitogen-activated protein kinase kinase kinase 3	Map3k3	−2.08	–	–	–	–
12	NM_011948	Mitogen-activated protein kinase kinase kinase 4	Map3k4	−1.87	−3.52	−1.98	1.51	–
13	NM_008580	Mitogen-activated protein kinase kinase kinase 5	Map3k5	−1.71		–	–	–
14	NM_016693	Mitogen-activated protein kinase kinase kinase 6	Map3k6	1.58	−5.25	3.25	–	–
15	NM_172688	Mitogen-activated protein kinase kinase kinase 7	Map3k7	1.51	−3.07	2.52	3.29	–
16	NM_025609	Mitogen-activated protein kinase kinase kinase 7-interacting protein 1	Map3k7ip1	–	−3.93	–	−1.91	–
17	NM_138667	Mitogen-activated protein kinase kinase kinase 7-interacting protein 2	Map3k7ip2	−1.56	**–**	**–**	**–**	**–**
18	NM_007746	Mitogen-activated protein kinase kinase kinase 8	Map3k8	−2.04	–	−0.58	2.76	1.55
19	NM_177395	Mitogen-activated protein kinase kinase kinase 9	Map3k9	–	−6.48	3.06	4.77	–
20	NM_009582	Mitogen-activated protein kinase kinase kinase 12	Map3k12	−7.07	−3.51	**–**	**–**	**–**
21	NM_016896	Mitogen-activated protein kinase kinase kinase 14	Map3k14	1.38	–	2.12	1.51	–
22	NM_008279	Mitogen-activated protein kinase kinase kinase kinase 1	Map4k1	–	−3.35	–	–	1.4
23	NM_009006	Mitogen-activated protein kinase kinase kinase kinase 2	Map4k2	−6.72		2.35	−1.35	–
24	NM_001081357	Mitogen-activated protein kinase kinase kinase kinase 3	Map4k3	−1.73		–	–	–
25	NM_008696	Mitogen-activated protein kinase kinase kinase kinase 4	Map4k4	−1.58	−3.54	2.46	–	−0.74
26	NM_201519	Mitogen-activated protein kinase kinase kinase kinase 5	Map4k5	−2.45	−3.28	3.94	−2.52	–
27	NM_031248	Mitogen-activated protein-binding protein-interacting protein	Mapbpip	2.29		–	–	–
28	NM_001038663	Mitogen-activated protein kinase 1	Mapk1	–	–	–	1.08	1.51
29	NM_011952	Mitogen-activated protein kinase 3	Mapk3 (ERK1)	−1.4	−7.48	–	–	1.09
30	NM_172632	Mitogen-activated protein kinase 4	Mapk4	–	−3.51	–	–	1.11
31	NM_015806	Mitogen-activated protein kinase 6	Mapk6	–	−3.53	2.11	1.76	1.07
32	NM_011841	Mitogen-activated protein kinase 7	Mapk7	−16.44	–	–	–	−0.99
33	NM_016700	Mitogen-activated protein kinase 8	Mapk8	–	–	10.39	12.43	1.21
34	NM_011162	Mitogen-activated protein kinase 8-interacting protein 1	Mapk8ip1	2.22	−10.62	–	–	−0.84
35	NM_016961	Mitogen-activated protein kinase 9	Mapk9	−1.88	−4.77	1.68	2.33	1.4
36	NM_009158	Mitogen-activated protein kinase 10	Mapk10	1.47	–	2.25	–	1.29
37	NM_011161	Mitogen-activated protein kinase 11	Mapk11	–	−10.9	1.78	2.32	–
38	NM_013871	Mitogen-activated protein kinase 12	Mapk12	–	–	−0.24	–	−0.88
39	NM_011950	Mitogen-activated protein kinase 13	Mapk13	1.44	–	–	−0.21	–
40	NM_011951	Mitogen-activated protein kinase 14	Mapk14	−1.62	–	1.14	−0.46	1.38
41	NM_145527	Mitogen-activated protein kinase-activating death domain	Mapkadd(Madd)	–	1.03			
42	NM_177345	Mitogen-activated protein kinase-associated protein 1	Mapkap1	−1.62	−6.56	−0.34	–	–
43	NM_008551	Mitogen-activated protein kinase-activated protein kinase 2	Mapkapk2	–	–	–	4.31	1.34
44	NM_178907	Mitogen-activated protein kinase-activated protein kinase 3	Mapkapk3	–	−3.39	–	2.38	1.19
45	NM_010765	Mitogen-activated protein kinase-activated protein kinase 5	Mapkapk5	–	−4.58	−0.42	−0.47	–
46	NM_011941	Mitogen-activated protein kinase binding protein 1	Mapkbp1	1.42	–	–	–	–
47	NM_010881	Nuclear receptor coactivator 1	Ncoa1	–	−3.55	–	–	–
48	NM_008679	Nuclear receptor coactivator 3	Ncoa3	–	–	−0.12	–	–
49	NM_144892	Nuclear receptor coactivator 5	Ncoa5	–	–	–	14.65	–
50	NM_172495	Nuclear receptor coactivator 7	Ncoa7	–	−4.46	–	–	–
51	NM_011308	Nuclear receptor corepressor 1	Ncor1	–	−5.37	3.39	–	–
52	NM_008689	Nuclear factor of kappa light chain gene enhancer in B-cells 1, p105	Nfkb1	−1.52	–	–	–	1.21
53	NM_019408	Nuclear factor of kappa light polypeptide gene enhancer in B cells 2, p49/p100	Nfkb2	−2.56	–	–	–	–
54	NM_010907	Nuclear factor of kappa light-chain gene enhancer in B-cell inhibitor, alpha	Nfkbia	–	–	–	–	−0.77
55	NM_030612	Nuclear factor of kappa light polypeptide gene enhancer in B cell inhibitor, zeta	Nfkbiz	–	–	–	2.67	–
56	NM_028024	NFKB inhibitor-interacting Ras-like protein 2	Nkiras2	−1.33	–	–	–	−0.94
57	NM_010902	Nuclear factor, erythroid-derived 2, like 2	Nrf2	1.54	–	–	–	1.26
58	NM_173440	Nuclear receptor-interacting protein 1	Nrip1	–	–	2.63	2.87	–
59	NM_020005	P300/CBP-associated factor	P/caf	–	–	–	2.33	–

BHA=butylated hydroxyanisole; EGCG=epigallocatechin-3-gallate; ER=endoplasmic reticulum; SFN=sulphoraphane.

a Fold-change values are listed.
